# Low-intensity educational interventions supporting self-management to improve outcomes related to chronic breathlessness: a systematic review

**DOI:** 10.1038/s41533-019-0152-8

**Published:** 2019-11-29

**Authors:** Bronwyn Raymond, Tim Luckett, Miriam Johnson, Ann Hutchinson, Melanie Lovell, Jane Phillips

**Affiliations:** 1 IMPACCT - Improving Palliative, Aged and Chronic Care through Clinical Research and Translation, Faculty of Health, University of Technology Sydney, 235-253 Jones Street, Ultimo, NSW 2007 Australia; 2 Hull York Medical School, University of York, John Hughlings Jackson Building, Heslington, York, Y010 5DD UK; 3 HammondCare, 95-115 River Road, Greenwich, NSW 2065 Australia; 4 Faculty of Medicine and Health, The University of Sydney, Science Road, Camperdown, NSW 2050 Australia

**Keywords:** Patient education, Quality of life

## Abstract

Chronic breathlessness is debilitating and frightening, often resulting in emergency department presentations with acute-on-chronic breathlessness. Self-management is complex, involving 14 components as identified by the Practical Systematic Review in Self-Management Support (PRISMS). Low-intensity educational interventions that support breathlessness self-management through written/visual educational materials, alongside limited health professional support, are available. Our aim was to describe components of low-intensity educational interventions that support and improve self-management for adults with chronic breathlessness and evaluate their efficacy for improving breathlessness-related outcomes. A systematic review was conducted, including RCTs that compared these interventions with usual care in adults with chronic disease. Synthesis took a narrative approach utilizing the PRISMS taxonomy and Template for Intervention Description and Replication (TIDieR) checklist. Of the 1948 articles identified, 7 met criteria reporting 7 RCTs using 6 interventions. Studies utilized 12 out of 14 PRISMS components, the most frequent being training/rehearsal for psychological strategies. Evidence for effectiveness was inconsistent and attempts to identify beneficial components were confounded by intervention complexity and heterogeneity. The optimal content and delivery of low-intensity educational interventions that support self-management to improve chronic breathlessness-related outcomes in adults cannot be defined from current published literature. Future research should incorporate more detailed, standardized reporting to enable comparison and meta-analysis.

## Introduction

Chronic breathlessness is a complex and incapacitating syndrome, which occurs commonly across a wide range of chronic conditions, especially chronic obstructive pulmonary disease (COPD), heart failure, and cancer.^1,2^ Experience of breathlessness is individual and multidimensional, encompassing sensory-perceptive, affective, and impact domains.^3^ Breathlessness restricts people’s everyday activities and social activities, while acute-on-chronic breathlessness^4^ can induce fear and panic, and worsening breathlessness is a reminder of disease progression and impending mortality.^5^

Living with chronic breathlessness requires a commitment to self-management and the ability to apply a range of pharmacological and non-pharmacological interventions to maintain emotional, social, and physical functioning.^6^ Self-management of chronic disease is a complex undertaking, with a recent taxonomy developed through a systematic review (Practical Reviews in Self-Management Support (PRISMS))^7^ identifying 14 components through which support can be provided. There is a great deal of interest in how best to support self-management of those with chronic breathlessness in a cost effective, accessible way,^6^ including how to help people self-manage acute-on-chronic breathlessness to avert avoidable emergency department presentations.^8^

Previously conducted systematic reviews have predominantly focused on interventions to improve self-management-related outcomes on COPD,^9–16^ some with a focus on efficacy of interventions that improve outcomes related to exacerbations^10,11,16^ and others on efficacy of intense training and support from a multidisciplinary team.^17,18^ Intensive interventions, such as pulmonary rehabilitation, have demonstrated effectiveness in improving patient activity,^19^ self-efficacy,^12,20^ health-related quality of life (HRQoL),^10^ total patient health, reducing patient burden,^19^ and hospital admission rates.^10,12–14^ To date, only two systematic reviews have focused on interventions not requiring substantial input from health professionals,^15,16^ despite the fact that many patients lack access to more resource-intensive services.^21,22^ These reviews did not synthesize effectiveness according to how strategies contributed to various self-management functions.

Consequently, we undertook a systematic review with the aims of: (1) describing how the PRISMS components of self-management have been addressed by low-intensity educational interventions to improve chronic breathlessness-related outcomes in adults and (2) evaluating which PRISMS components are present in interventions that have been shown to be effective.

## Results

### Study selection

**Fig. 1:**
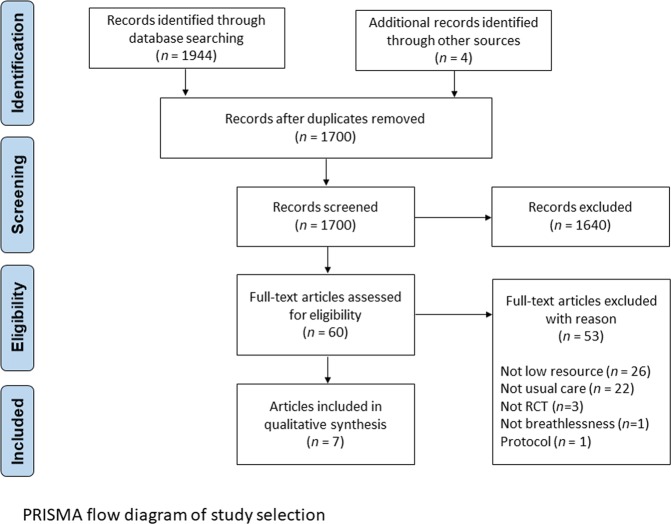
PRISMA flow diagram of the study selection. This schematic shows a flowchart depicting the process of study selection and exclusion. RCT randomized controlled trial.

Searches identified 1948 articles (including 4 hand-searched articles), of which 7 met eligibility criteria (Fig. 1).^23–28^

### Characteristics of the included studies

All seven studies employed parallel designs, two of which utilized the same intervention.^25,27^ All except one study included patients with COPD with/without other conditions, with one focusing on intrathoracic malignancies.^28^ Four of the eight studies recruited patients with a diagnosis of COPD, either diagnosed using an international standard or the acceptance of a doctor’s diagnosis.^23,25–27^ Of the other three studies, two included patients with chronic conditions such as chronic heart failure/cardiovascular disease, diabetes, hypertension, arthritis, chronic respiratory conditions, and palliative patients,^24,29^ the other included patients with intrathoracic malignancies.^28^ Patients were predominantly recruited from hospital inpatient and outpatient clinics,^23,25–29^ except in one study that recruited through established websites and discussion groups.^24^ The interventions were chiefly conducted in hospital clinics or laboratories,^23,28,29^ two were carried out in the general practitioners’ surgery^25,27^ and a further two were performed online.^24,26^ The characteristics of each study are summarized in Table 1.

**Table 1:** Characteristics of the included RCTs.

Reference and country	Sample (*N*)	Mean age (SD)/gender (% female)	Condition of interest	Other notable characteristics	Randomized control trial—evaluative	Primary endpoint	Breathlessness-related outcome measures
Hochstetter et al.;^29^ UK	31 Con 15 Int 15	76 Con (15.4) Int (15.6)/ Con 33% Int 53%	CVD, palliative, surgical, respiratory pathology+breathlessness climbing stairs	Abbreviated Mental Test >6; inpatient 3 consecutive days; independently mobile; no physiology for SOB; medically stable; English language	*N* = 13 at 90% (*p* = 0.05) was required	Day 3	Adapted BORG^a^ Top stairs Base stairs Number of stops
Johnson et al.;^28^ UK	156 Con 52 Int 104	69 Con (11) Int (9)/ Con 21% Int 39%	Intrathoracic malignancy	Refractory breathlessness with a self-reported intensity of ≥3/10 on the NRS	*N* = 146 powered at 80% for a 2-tailed *p* = 0.05	1 month	NRS (Breathlessness severity) Intensity^a^ Average intensity; coping Distress CRQ-SAS HRQoL Dyspnea domain; fatigue; emotional function; Mastery HADS
Lorig et al.;^24^ USA/UK	958 Con 501 Int 457	58 Con (11.3) Int (10.5)/ Con 72% Int 71 %	Heart or chronic lung disease, type II diabetes, hypertension, and arthritis	>18 years; no active cancer treatment for 1 year	Randomization checked using *t* test comparing baseline demographics	12 months	VNS^a^ (Symptom Severity) SOB, pain, fatigue, health distress Chronic Disease Efficacy Scale Self-Efficacy
McGeoch et al.;^25^ NZ	159 Con 73 Int 86	71 Con (9.9) Int (11.6)/ Con 33% Int 48%	COPD (American Thoracic Society criteria)	Forced expiratory volume/forced vital capacity <70%; ≥1 exacerbation in last year needing increase in therapy; not using self-management plan or domiciliary oxygen	*N* = 80 powered at 80% for a 2-sided alpha (*p* = 0.05)	6 months	SGRQ^a^ HRQoL ADS COPD-SMI Well knowledge; well actions; early exacerbation knowledge; early exacerbation action; severe exacerbation knowledge; severe exacerbation action
Rootmensen et al.;^23^ Netherlands	191 Con 94 Int 97	60 Con (15) Int (15)/ Con 37% Int 46%	COPD or asthma	>18 years, no previous consult with respiratory nurse, Dutch speaking	*N* = 65 powered at 80% for a 2-sided alpha (*p* = 0.05)	6 months	SGRQ HRQoL Knowledge Inhalation Technique Self-management Questionnaire Exacerbation
Voncken-Brewster et al.;^26^ Netherlands	1325 Con 663 Int 662	58 Con (7.2) Int (7.3)/ Con 51% Int 54%	COPD or moderate to high risk of COPD	40–70 years of age; Dutch speaking	*N* = 446 powered at 80% for 10 min diff. at *p* = 0.05	6 months	MRC Dyspnea Scale (Breathlessness Severity) CCQ – Clinical Disease Control
Watson et al.;^27^ NZ	69 Con 27 Int 29	68 Con (8) Int (10)/ Con 33% Int 38%	COPD	Smoking history of >10 pack years; FEV1 < 65% predicted; FVC < 70%; currently using bronchodilator therapy	*N* = 166 power of 0.8 for *p* = 0.05 to detect a change of 5.7 points in the total score of the SGRQ	6 months	SGRQ^a^ HRQoL Diary for Respiratory symptoms

*Adapted BORG* Adapted BORG Scale of Perceived Exertion, *Con* Control *CCQ* Clinical COPD Questionnaire, *COPD* Chronic obstructive pulmonary disease, *CRQ-SAS* Chronic Respiratory Questionnaire-Self-administered Survey, *COPD-SMI* Chronic Obstructive Pulmonary Disease–Self Management Interview, *CVD* Cardiovascular disease, *HADS* Hospital Anxiety Depression Scale, *HRQoL* Health Related Quality of Life, *Int* Intervention, *MRC* Medical Research Council Dyspnea Scale, *NRS* Numeric Rating Scale, *SGRQ* St George Respiratory, *RCT* randomized controlled trial *SOB* shortness of breath

^a^Primary outcome

### Risk of bias in the individual studies

An overview of the risk of bias for the included studies is provided in Table 2.

**Table 2:** Cochrane risk of bias summary.

Included studies	Random sequence generation	Allocation concealment	Performance bias	Detection bias	Attrition bias	Other bias
Hochstetter et al.^29^	Low risk	High risk	High risk	High risk	Low risk	Low risk
Johnson et al.^28^	Low risk	Low risk	High risk	Low risk	Low risk	Low risk
Lorig et al.^24^	Unclear	Unclear	Unclear	Unclear	Low risk	Low risk
McGeoch et al.^25^	Low risk	High risk	High risk	High risk	Low risk	High risk
Rootmensen et al.^23^	Low risk	Low risk	Low risk	Low risk	Low risk	Low risk
Voncken-Brewster et al.^26^	Low risk	Low risk	Low risk	Low risk	Low risk	Low risk
Watson et al.^27^	Unclear	Unclear	Unclear	Unclear	Low risk	Low risk

This Cochrane risk of bias summary table indicates that the most common risks of bias in the review were a lack of allocation concealment and blinding

### Synthesis

#### Aim 1: To describe how the PRISMS components of self-management have been addressed by low-intensity educational interventions to improve chronic breathlessness-related outcomes

##### PRISMS taxonomy

Twelve of the 14 components of the PRISMS taxonomy^7^ were utilized to varying degrees, with the number of times any one component was addressed varying from once (*(A2) Information on available resources*)^26^ to four times (*(A12) Training and rehearsal of psychological strategies*).^23,24,26,28^ One intervention utilized only two components^29^ while one incorporated seven components.^24^ Two components were not addressed by any of the included interventions (*(A4) A regular clinical review*; *(A7)* *The provision of equipment to enable/promote self-monitoring and/or self-management*), see Table 3 for a breakdown by study.

**Table 3:** Study characteristics and breathlessness-related outcomes.

Study	Intervention—PRISMS taxonomy items^7^														Staff	Modality				Group versus 1:1			Freq.	HP	Positive breathlessness-related outcomes	No effect breathlessness-related outcomes
	(A1) Information—condition and management	(A2) Information on available resources	(A3) Provision of specific clinical action plans+/−rescue medication	(A4) Regular clinical reviews	(A5) Monitoring of condition with feedback	(A6) Practical support with adherence	(A7) Provision of equipment	(A8) Provision of easy access to advice or support	(A9) Training/rehearsal to communicate with health-care staff	(A10) Training/rehearsal for everyday activities	(A11) Training/rehearsal—practical self-management activities	(A12) Training/rehearsal for psychological strategies	(A13) Social support	(A14) Lifestyle advice and support		Handout	Web based	Face-to-face	Other (video or phone call)	Group	1:1	Individualized				
Hochstetter et al.^29^	✓	—	—	—	—	—	—	—	—	✓	✓	—	—	—	Physiotherapist	✓	—	✓	—	—	✓	✓	×1	45 min^a^	Adapted BORG Top stairs (*p* = 0.002) Base stairs (*p* = < 0.001) Stops I only (*p* = 0.003)	
Johnson et al.^28^	—	—	—	—	—	—	—	—	—	✓	✓	✓	—	—	Therapist	✓	—	✓	✓	—	✓	✓	×1	60 min	NRS (Breathlessness severity) Distress only (*p* = 0.01, d = 0.509) CRQ-SAS Mastery only (*p* = 0.02, *d* = 0.491)	NRS Worst and average breathlessness
Lorig et al.^24^	✓	—	—	—	—	✓	—	✓	✓	—	—	✓	✓	✓	Trained mod.	✓	✓	—	—	✓	—	✓	Ad Lib	Nil	VNS SOB (*p* = 0.02, d = 0.229), Pain (*p* = 0.011, *d* = 0.032), Fatigue (*p* = 0.04, *d* = 0.151), Health Distress (*p* = 0.25, *d* = 0.160)	Self-Efficacy Scale
McGeoch et al.^25^	—	—	✓	—	✓	—	—	✓	✓	—	—	—	—	—	Nurse	✓	—	✓	—	—	✓	✓	×1	60 min	COPD-SMI WK (*p* = 0.001), EEK (*p* = 0.001) EEA (*p* = 0.001), SEK (*p* = 0.002) SEA (*p* = 0.005)	SGRQ HRQoL HADS
Rootmensen et al.^23^	✓	—	—	—	✓	✓		✓	—	—	—	✓	—	✓	Nurse	—	—	✓	—	—	✓	✓	×1	45 min	Exacerbation (*p* = 0.04, OR = 0.35)	SGRQ HRQoL Knowledge IT SMQ
Voncken-Brewster et al.^26^	✓	✓	—	—	✓	—	—	—	—	—	—	✓	✓	✓	NA	—	✓	—	✓	—	—	✓	Ad Lib	NS		MRC breathlessness severity CCQ
Watson et al.^27^	—	—	✓	—	—	—	—	✓	✓	✓	—	—	—	✓	Nurse	✓	—	✓	✓	—	✓	✓	×1	60 min	DFRS Initiate steroid (*p* = 0.014) Initiate Abs (*p* = 0.002) Days on Abs (*p* = 0.016, *d* = 0.677)	SGRQ HRQoL Days on steroids

The table describes study characteristics including the 14 components of the PRISMS taxonomy and positive and negative breathlessness-related outcomes

*Adapted BORG* Adapted BORG Scale of Perceived Exertion, *Ad Lib* Ad Libitum, *Abs* antibiotics, *CCQ* Clinical COPD Questionnaire, *CRQ-SAS* Chronic Respiratory Questionnaire-Self-administered survey, *COPD-SMI* Chronic Obstructive Pulmonary Disease-Self Management Interview, *d* Cohen’s *d* *DFRS* Diary for respiratory symptoms, *EEA* Early Exacerbation Action, *EEK* Early Exacerbation Knowledge, *HADS* Hospital Anxiety and Depression Scale, *HRQoL* Health Related Quality of Life, *EF* Emotional Function, *IT* Inhalation Technique, *HP (min)* health support in minutes, *MRC* Medical Research Council Dyspnea Scale, *NA* not applicable, *NS* not specified, *OR* odds ratio, *P* Physiotherapist, *p* level of significance <0.05, *RCT* randomized controlled trial, *SEK* Severe Exacerbation Knowledge, *SEA* Severe Exacerbation Action, *SMQ* Self-Management Questionnaire, *mod* moderator, *SGRQ* St George Respiratory Questionnaire, *WA* weak actions *WK* weak knowledge

^a^Breathing technique taught as per individual study protocol

Four interventions instructed patients on their *condition and its management (A1)* either through scripted information or answering patient questions.^23^^,24,26,30^ Interventions by Hochstetter et al.^29^ and Rootmensen et al. explained the physiological rationale underpinning the skills being taught^23,30^ while another by Voncken-Brewster et al. provided information on risk factors.^26^ Both the intervention by Voncken-Brewster et al. and another web-based program by Lorig et al. included information on the medical condition and its management.^24,26^ However, the intervention by Voncken-Brewster et al. differed in that it was exclusively an online program called “MasterYourBreath,”^26^ while the intervention by Lorig et al. also gave participants a hard copy of the book called “Living a Healthy Life with Chronic Conditions.”^24^ These interventions provided access to websites and videos designed to assist the patient in seeking out further information, while only the intervention by Voncken-Brewster et al. gave *information on available resources (A2)* in the form of advice on where to connect with peer and social support.^26^

The intervention evaluated by both Watson et al. and McGeoch et al. was the only one to implement an action plan (*specific clinical action plans with medications (A3)*), which provided guidance for patients in recognizing and acting on early signs of exacerbation (for example, the use of rescue medication).^25,27^

Of the three interventions that *monitored the patient’s condition with feedback* *(A5)*, one gave patients nonspecific information with regards to self-monitoring their condition, which was not elaborated on,^23^ while another gave feedback with regards to behavior and motivation through the use of a diary.^26^ An action plan intervention by Watson et al. and McGeoch et al. encouraged patients to observe changes in sputum color to warn of infection.^25,27^

The interventions by Lorig et al. and Rootmensen et al. included practical *support with adherence* *(A6)*, both of which checked inhalation technique and the proper use of medications specifically.^23,24^

Of the three interventions that *provided easy access to advice and support* *(A8)*, one intervention allowed patients to email questions to the moderator of their online program, another gave special access to call a doctor or nurse,^23^ and the action plan intervention used by two studies provided patients with the number for the ambulance and support services as a prompt for action when in a distressed state.^25,27^

To assist patients’ interactions with doctors and nurses, *training and rehearsal to communicate with health professionals (A9)* was provided by two interventions. The action plan interventions by Watson et al. and McGeoch et al. gave patients information cards that contained clinical information (for example, forced expiratory volume) for health professionals,^25,27^ while the intervention by Lorig et al. supported “aspects of patient physician communication” without specifying further.^24^

Three interventions incorporated *training and rehearsal for everyday activities (A10)* by providing information to simplify daily activities^25,27^ and practice pacing when climbing stairs to control breathing.^28,29^

Two interventions focused on breathing techniques as *training and rehearsal for practical self-management activities (A11)*.^28,29^ These included controlled breathing techniques by Hochstetter et al.^29^ and positioning for breathing control by Johnson et al.^28^

*Training and rehearsal for psychological strategies (A12)* was represented in four interventions and included: a five-step behavioral change program including training and motivation to set goals and carry out a plan;^26^ an extensive problem-solving and decision-making program with cognitive symptom management (relaxation, visualization, distraction, self-talk, methods for managing negative emotions, such as fear, anger, depression, and frustration);^24^ progressive muscle relaxation and anxiety management through the use of a tool “The Calming Hand”;^28^ and instructions for the patients on how to remain calm in the case of acute-on-chronic breathlessness.^23^

*Social support (A13)* was encouraged by two interventions: Lorig et al. provided online bulletin boards,^24^ Voncken-Brewster et al. provided information on how to cope with social pressures.^26^

*Lifestyle support (A14)* was provided by four interventions in the form of encouragement to cease smoking;^23,25–27^ education in aspects of eating;^24,27^ fatigue management and exercise advice;^24^ the importance of avoiding allergens, triggers for breathlessness, and vaccinations.^23^

Self-management interventions were employed using diverse modalities: five studies utilized face-to-face methods;^23,25,27–29^ five utilized written handout material;^24,25,27–29^ two studies utilized online/web-based systems;^24,26^ and three studies utilized either a phone call, video/DVD, or both.^26–28^ Four studies utilized two modalities,^24–26,29^ another two studies utilized three modalities,^27,28^ and a further study utilized a solo modality.^23^ Interaction with patients was group, via internet-based chat rooms,^24^ or one-on-one.^23,25–29^

#### Aim 2: To evaluate which PRISMS components are present in interventions that have been shown to be effective

Five interventions (six studies) demonstrated efficacy in improving at least one breathlessness-related outcome, although results were inconsistent across outcomes (see Table 3).

The study by Johnson et al.^28^ focusing on intrathoracic malignancies compared a one-off session utilizing breathing management techniques (breathing control, pacing/prioritizing, relaxation, and anxiety management) versus a control arm that included three sessions. The single session was not found to be inferior to three sessions in reducing worst breathlessness intensity scores over 24 h (*p* = 0.83). A single session resulted in a significant moderate improvement of patients’ sense of mastery over breathlessness on the Chronic Respiratory Questionnaire-Self-administered survey (*p* = 0.02, *d* = 0.491) and reduction in distress due to breathlessness (*p* = 0.01, *d* = 0.509).^28^

The internet-based, comprehensive self-management program by Lorig et al.^24^ for patients with a range of chronic diseases identified significant improvements of small effect size in levels of fatigue (*p* = 0.04, *d* = 0.151), health distress (*p* = 0.025, *d* = 0.160), shortness of breath (*p* = 0.02, *d* = 0.229) and pain (*p* = 0.011, *d* = 0.032), and self-efficacy trended toward significance (*p* = 0.061, *d* = 0.096) at 12 months. Patients were also found spending significantly more time each week undertaking stretching and strengthening exercises (*p* = 0.024).^24^

The intervention by Hochstetter et al.,^29^ which utilized physiotherapy-based breathlessness management including teaching diaphragmatic and pursed lip breathing techniques, demonstrated a significant reduction in reported breathlessness when using the adapted BORG scale of Perceived Exertion at the top (*p* = 0.002) and bottom of the stairs (*p* < 0.001). Patients in the intervention group also significantly increased the number of stops they made on day 3 of the intervention (*p* = 0.003).^29^

Rootmensen et al.^23^ found a protocol-based education program to reduce causes of chronic breathlessness and teach the correct actions to take regarding medication usage, triggers, self-monitoring, smoking cessation, and individual self-management instructions. The intervention led to a significant moderate reduction in frequency of exacerbations (*p* = 0.04, odds ratio (OR) = 0.35).^23,31^

Watson et al.^27^ taught patients to use an action plan that changed medications usage. The intervention increased the number of patients who self-initiated medication when their condition deteriorated: prednisone (*p* = 0.014) and antibiotics (*p* = 0.002) following worsening symptoms, for example, color change in sputum. The number of days patients were on antibiotics increased in the intervention group (*p* = 0.016; *d* = 0.677).^27^ McGeoch et al. found the same action plan to result in improvements on the COPD Self-Management Interview in relation to self-management knowledge leading to feelings of wellness, early exacerbation knowledge and actions (*p* = 0.001), and severe exacerbation knowledge (*p* < 0.005) and actions (*p* = 0.005).^25^

No clear patterns were discernible based on classifying interventions using either the PRISMS or Template for Intervention Description and Replication (TIDieR) frameworks. However, upon investigation of interventions through the lens of the PRISMS framework, the frequency of components present in effective interventions can be considered. *Easy access to support (A8)*^23–25,27^ was present in 38% (*n* = 3) and 44% (*n* = 4) of the included interventions and studies demonstrating positive outcomes, respectively. *Information on disease (A1)*,^23,24,29^ *Training/rehearsal for everyday activities (A10)*,^27–29^ *Training/rehearsal for practical self-management activities (A11)*,^28,29^ *Lifestyle (A14)*,^23,24,27^ and *Psychological support (A12)*^23,24,28^ were each present alongside favorable outcomes in 38% (*n* = 3) and 33% (*n* = 3) of interventions and studies, respectively. *Training/rehearsal for communication with health care professionals (A9)*^24,25,27^ was present in 25% (*n* = 2) and 33% (*n* = 3) of interventions and studies finding beneficial results, respectively. *Monitoring of condition with feedback (A5)*^23,25^ and *Practical support with adherence (A6)*^23,24^ were present alongside effective outcomes in 25% (*n* = 2) of interventions and 22% (*n* = 2) of studies. Finally, *Provision of specific clinical action plans+/−resource medications (A3)*^25,27^ and *Social Support (A13)*^24^ were each present in only one intervention found to be effective. The intervention that included *provision of specific clinical action plans*+*/−resource medications (A3)* was found to be efficacious in two studies.^25,27^

Successful interventions varied in terms of modality, individual versus group focus, degree of tailoring, and personnel involved in delivery. Face-to-face delivery was used in five efficacious interventions,^23,25,27–29^ with one of these also using a telephone call,^27^ and another using both a telephone call and a DVD.^28^ Handouts were used in five effective interventions,^24,25,27–29^ with two others using a DVD^27^ and internet,^24^ and four using combined modalities.^25,27–29^

Interventions delivered one-on-one by a health professional were more often present alongside beneficial outcomes than group interventions.^23,25,27–29^ These efficacious interventions were tailored to individual patient needs.^23–25,27–29^ Nurses were the most frequently utilized personnel (*n* = 3);^23,25,27^ however, two of these studies also included medical doctors who wrote scripts for medications such as steroids and antibiotics required by the action plan.^25,27^

## Discussion

Low-intensity educational interventions to improve the self-management of chronic breathlessness in adults identified in this review were predominantly focused on COPD and multicomponent in nature, as defined by the PRISMS Taxonomy.^7^ Collectively, interventions (12 out of 14) offered comprehensive coverage of self-management components, although some components were addressed more consistently than others. Evidence for efficacy was mixed, and no single component or intervention characteristic could be isolated as contributing to improved breathlessness or related outcomes to inform future practice.

This current review aligns with previous systematic reviews,^1,2,9,16,32,33^ which found evidence that training and rehearsal of practical self-management activities can positively reduce distress and physiological measures of breathlessness, including pursed lip breathing, exercise, positioning, and pacing. In the current review, both Johnson et al. and Lorig et al. used validated numeric rating scales, which identified improvement in health-related distress by a moderate and small effect size, respectively,^24,28^ following low-intensity educational interventions. A systematic review by Cannon et al. found that interventions of <5 weeks’ duration significantly improve symptom and activity outcomes.^12^ Our review adds that these strategies can sometimes improve outcomes with an even lower level of support from health professionals. Johnson et al. provided the clearest demonstration of this by showing one 60-min intervention session to be at least as effective and cost-effective as three 60-min programs, with lower burden on patients and the health system.^28^

This review included three of the five low-intensity studies^23,25,27^ from a Cochrane review investigating the effectiveness of action plans on breathlessness,^16^ as two interventions did not state the duration of time spent with health professionals. The three studies found evidence for improvements in patient knowledge in recognizing a deteriorating condition and prompting appropriate initiation of medications. However, other reviews that have evaluated the use of action plans within the context of resource-intensive interventions have found that these can also improve HRQoL and reduce hospital use,^9,10^ suggesting that health professional support may boost efficacy.

This review found mixed evidence for improving self-efficacy, with one intervention finding an improvement in breathlessness mastery,^28^ while an observed benefit in a second study did not reach statistical significance.^24^ Self-efficacy is important as chronic breathlessness is known to increase one’s vulnerability to emotional influences and lead to a perceived loss of control.^34^ A coaching approach that acknowledges the social context of self-efficacy may be an important element that is difficult to include in low-intensity education interventions with minimal health professional support.^6,35^ Also, higher levels of self-efficacy have been associated with patients’ ability to ask questions of their health-care providers,^36^ suggesting that there may be a “virtuous cycle” where health professionals are involved. The interaction between a patient’s coping and help-seeking approach, and their health-care professional’s attention to breathlessness as a symptom in addition to disease management, has also been described as influencing a patient’s ability to live well with chronic breathlessness (a concept known as Breathing Space).^37^

Several components of self-management as defined by PRISMS were not well addressed by the low-intensity education interventions included in this review. Setting aside regular clinical review and use of equipment, which require a high level of health professional supervision specifically excluded by our criteria, it is unclear why only one intervention included information on available resources. Social support was addressed in only two interventions in the current review, neither of which measured social outcomes.^24,26^ Both were internet-based programs limited either by small participant numbers^24^ or low uptake by participants.^26^ Variable participation is not uncommon in e-health interventions, suggesting that this modality may better suit certain populations.^15,38^ Further research is needed to evaluate other modalities of providing social support for people with breathlessness and their carers, including face-to-face and telephone.^35,39^ A better understanding of how to optimize the influence of social support through patient-tailored self-management interventions could considerably impact breathlessness-related outcomes.^39^

Similar to other reviews, these findings are limited by heterogeneity among self-management programs for breathlessness.^1,9,14,15^ Our attempt to use two frameworks to “unpack” this heterogeneity was hampered by a lack of reported detail, commonly encountered by reviews of complex interventions across health conditions and settings.^40^ Although no clear patterns were discernible following classification of interventions by the PRISMS taxonomy^7^ or the TIDieR framework, it remains possible that a combination of content and delivery produced synergistic effects. More interventions might have been included in this review had more detailed information been included concerning the duration of health professional involvement. Authors reporting future studies are encouraged to utilize frameworks like the PRISMS and TIDieR to ensure sufficient information about interventions is provided in a way that enables comparison of studies.

In conclusion, there is mixed evidence that low-intensity educational interventions for the support of self-management of chronic breathlessness in adults can lead to improvements in breathlessness, functioning, or breathlessness-related HRQoL. Overall, evidence for efficacy is more limited for low-intensity interventions than for higher-intensity interventions that include ongoing support from a health professional. Further investigation is needed to identify the most effective components and modalities of low-intensity self-management education to improve patients’ chronic breathlessness and the optimal cost-effectiveness of combining these with health professional support.

## Methods

The systematic review was registered with the international prospective register of systematic reviews PROSPERO (registration number CRD42018108810).

### Eligibility criteria

Studies were included if they were randomized controlled trials published in English-language peer-reviewed journals that compared usual care with a low-intensity educational intervention aimed at supporting self-management of chronic breathlessness in adults. We defined low intensity as written, audio, or visual education/training materials supported by no more than 60 min (in total) of consultation with a health professional. Studies that did not identify the duration of health professional input were excluded. Interventions could be administered in any modality and location, provided they were intended to support patients to self-manage in the community setting. Interventions could focus on teaching aspects of self-managing breathlessness either on a day-to-day basis or during an acute-on-chronic episode. Participants could have chronic breathlessness due to any health condition (for example, lung disease, heart failure, cancer), except for a primary diagnosis of asthma, which was excluded because of its specific acute management needs. Studies needed to measure effectiveness of interventions on one or more dimensions of breathlessness or another symptom that may be related as evidenced by the fact that it commonly co-presents in symptom clusters, namely anxiety, depression, fatigue, sleeping difficulties, and pain.^41,42^

### Information sources

A search was conducted from inception of the database to March 2018 of electronic databases, including CENTRAL, Medline, and CINAHL. Reference lists of included studies and the systematic reviews referenced above were searched manually. This search was updated using the same search terms on 1 August 2019.

### Search terms

The search strategy was derived from a Cochrane review protocol by Howell et al., which included MeSH terms for breathlessness together with self-management or patient education.^43^ Strategies were modified for individual databases. Searches were limited to English language, humans, and aged >18 years (see Supplementary Information for search criteria).

### Study selection

The first 10% of search results were reviewed independently against eligibility criteria by two researchers (B.R. and T.L.), based on title/abstract and full text as needed. Following agreement of >95%, one researcher reviewed articles alone.

### Data collection and analysis

Data were extracted by a single researcher using an electronic proforma (B.R.), with random data checks performed by a second researcher (T.L.). Data items included: authors and date, country, aims, sample characteristics (diagnoses, age, gender), setting, intervention, comparator group, outcome measures, and statistical outcomes (see Tables 1 and 3).

Outcomes of interest measured in this systematic review are detailed in Supplementary Information.

Efficacy of interventions was determined by assessing the statistical significance (at a *p* < 0.05 level) and clinical significance (using effect size) of outcomes. Where effect sizes were reported or able to be calculated from included studies, we have reported these as Cohen’s *d* or OR. These were interpreted using the rule of thumb proposed by Cohen that 0.2 standard deviation (SD) be considered a “small” effect size, 0.5 a “medium” effect size, and 0.8 a “large” effect size and equivalent values for ORs.^30,31,44^

If any inconsistencies within studies were found, these were interpreted with reference to differences in the constructs and the measures involved.

Worsening respiratory symptoms in COPD are often due to exacerbations.^23^ Exacerbation encompasses the whole range of respiratory symptoms, for example, mucus and cough as well as breathlessness, which frequently requires prescribed oral steroids and/or antibiotics as treatment and is not a widely used term for other causes of chronic breathlessness. Therefore, in this review we use the term *acute-on-chronic breathlessness* to clarify the symptom under study, including episodic breathlessness (triggered or untriggered), and to allow other causes of breathlessness (such as heart failure and neuromuscular conditions) to be included.^4,45^

### Risk of bias within studies

Two researchers (B.R., T.L.) independently utilized the Cochrane risk of bias tool to assess studies as “high risk,” “low risk,” or “unclear’ across seven domains.^46^ Disagreements were resolved by discussion.

### Synthesis

Intervention components were classified using the PRISMS taxonomy^7^ and the TIDieR checklist.^47^ Classifications were conducted independently by two researchers (B.R., T.L.) who discussed as necessary to reach agreement. Synthesis took a narrative approach based on methods described by Popay et al., after it became clear that most interventions and measures were too heterogeneous to allow for meta-analysis.^48^

### Reporting summary

Further information on research design is available in the Nature Research Reporting Summary linked to this article.

## Supplementary information

Supplementary Information

Reporting summary

## Data Availability

The authors declare that the data supporting the findings of this study are available within the paper and its supplementary item files.
