# Supplementary material for: Low-intensity educational interventions supporting self-management to improve outcomes related to chronic breathlessness: a systematic review
Source: NPJ Prim Care Respir Med. 2019 Nov 29;29:41. doi: 10.1038/s41533-019-0152-8 (PMC6884574; doi:10.1038/s41533-019-0152-8)
Supplement: Supplementary file 1 — Supplementary Information [file 41533_2019_152_MOESM1_ESM.pdf]

Supplementary Item 1 - Search Strategy

The search strategy was derived from a Cochrane review protocol on the topic of interest (Howell et al., 2012) and included terms as follows:

Medline

1. Exp Dyspnea/ or Dyspn*.mp

2. breathless*.mp

3. (breath*adj5 (difficult* or labor* or labour* or discomfort or uncomfortabl* or distress*)).mp

4.(short*adj5 breath*).mp

5. 1 or 2 or 3 or 4

6. exp Self Care/

7. (self-manag* or selfmanag* or self-car* or selfcar* or self-monitor* or selfmonitor* or self-administrat* or selfadministrat* or self-medicat* or selfmedicat*).mp

8. exp Patient Education as Topic/

9. Pulmonary rehabilitation*.mp

10. 6 or 7 or 8 or 9

11. 5 and 10

12 exp Pediatrics/

13 exp Child/

14. 12 or 13

15. 11 not 14

16. limit 15 to (English language and humans)

CINAHL

S1	(MH “Dyspnea+”) OR “Dyspn*”
S2	(MH “Self Care+”) OR “Self Care”
S3	(MH “Symptom Distress”)
S4	(MH “Self Administration+”)
S5	(MH “Patient Education+”)
S6	(MH “Rehabilitation, Pulmonary+”)
S7	S2 OR S3 OR S4 OR S5 OR S6
S8	S1 and S7
S9	S8 Not Child Not Pediatric Limiters – Publication date19610101-20180331; English language

Supplementary Item 2 - Outcomes of Interest

Instrument	Description	Reference
**Adapted BORG Scale**	Measures subjective breathlessness during physical activity.	1
**Inhalation Technique**	An ad hoc assessment using videotape scored by trained observers using a device-specific inhalation checklist.	2
**Self-Efficacy Scale**	Measured perceived Self-efficacy which reflects patient's ability to manage condition.	3
**Chronic Respiratory Questionnaire Self-Administered-Survey (CRQ-SAS)**	assesses health-related quality of life in respiratory conditions.	4
**Clinical COPD Questionnaire (CCQ)**	Consists of 10 items (each scored between 0 & 6), divided into three domains (symptom, functional, mental).	5
**COPD Self-Management Interview (COPD-SMI)**	Descriptions read to subjects based on three stages of an exacerbation (feeling well, early exacerbation & severe exacerbation). In each scenario, three self-management domains are assessed: medication use, healthcare seeking decisions and self-care.	6
**Diary for respiratory symptoms**	Patients rated their respiratory status on a four-point scale, using the categories: usual, mild, moderate or severe. The diary card recorded: prednisone & antibiotic use; contact with doctors, nurses, specialist, pharmacist & other comments.	7
**Exacerbation**	An ad hoc questionnaire designed to collect patient's exacerbation event as judged by medical officer to require a prescription for oral steroids.	8
**Hospital Anxiety and Depression Scale (HADS)**	Measures anxiety & depression in the medical setting	9
**Medical Research Council Scale (MRC) Dyspnoea Scale**	Assesses breathlessness severity with regard to the level of disability caused.	10
**Number of Stops**	An ad hoc measure that counts the number of stops required by patients to avoid breathlessness during physical activity.	11
**Numeric Rating Scale (NRS)**	used to measures patient-reported intensity of the worst & average breathlessness over the past 24 hours ('breathlessness severity'), 'distress' & 'coping' with breathlessness.	1, 12
**Self-administered Knowledge Questionnaire**	An ad hoc questionnaire designed by the study team with 18 items formulated by respiratory physicians & nurses	2
**Self-management Questionnaire**	Uses two rapid onset & two slow onset scenarios of exacerbations adapted from a validated qualitative questionnaire	13
**St George Respiratory Questionnaire (SGRQ)**	A disease-specific quality of life, measuring- 'Symptom' (distress due to respiratory symptoms), 'Activity' (effect on mobility & physical activity), & 'Impact' (assesses impact of disease).	14
**Visual Numeric Scales (VNS)**	adapted from the Visual Analogue Scale (VAS), was used to measure 7 HRQoL health indicators.	15

Supplementary item 2 describes the breathlessness-related outcomes utilised in the systematic review and narrative synthesis entitled "*Low-intensity educational interventions supporting self-management to improve outcomes related to chronic breathlessness a systematic review".* Whilst some outcomes are not directly measuring breathlessness, they do have breathlessness-related dimensions as identified in our introduction – i.e. sensory-perceptive, affective or impact. These domains are featured in HRQoL measures such as the St George Respiratory Questionnaire (SGRQ). Some other symptoms – most notably anxiety, fatigue, insomnia and pain – commonly present with breathlessness as a cluster. Indeed, while the HADS ostensibly measures anxiety and depression in a generalised way, measurement of these constructs in people with chronic breathlessness is likely to at least in part be assessing the affective dimension of breathlessness.

References:

1 Wilson, R. C. & Jones, P. A comparison of the visual analogue scale and modified Borg scale for the measurement of dyspnoea during exercise. *Clinical Science* **76**, 277-282 (1989).

2 Rootmensen, G. N. *et al.* The effects of additional care by a pulmonary nurse for asthma and COPD patients at a respiratory outpatient clinic: Results from a double blind, randomized clinical trial. *Patient Education and Counseling* **70**, 179-186, doi:10.1016/j.pec.2007.09.021 (2008).

3 Lorig, K., Ritter, P., Laurent, D. & Plant, K. Internet-Based Chronic Disease Self-Management: A Randomized Trial. *Medical Care* **44**, 964-971 (2006).

4 Schünemann, H. *et al.* A randomised trial to evaluate the self-administered standardised chronic respiratory questionnaire. *European Respiratory Journal* **25**, 31-40 (2005).

5 Kon, S. S. C. *et al.* The Clinical COPD Questionnaire: response to pulmonary rehabilitation and minimal clinically important difference. *Thorax* **69**, 793, doi:10.1136/thoraxjnl-2013-204119 (2014).

6 Dowson, C. A., Town, G. I., Frampton, C. & Mulder, R. T. Psychopathology and illness beliefs influence COPD self-management. *Journal of psychosomatic research* **56**, 333-340 (2004).

7 Watson, P. B. *et al.* Evaluation of a self-management plan for chronic obstructive pulmonary disease. *Eur Respir J* **10**, 1267 (1997).

8 Pauwels, R. *et al.* COPD exacerbations: the importance of a standard definition. *Respiratory Medicine* **98**, 99-107, doi:10.1016/j.rmed.2003.09.001 (2004).

9 Snaith, R. P. The hospital anxiety and depression scale. *Health and quality of life outcomes* **1**, 29 (2003).

10 Bestall, J. *et al.* Usefulness of the Medical Research Council (MRC) dyspnoea scale as a measure of disability in patients with chronic obstructive pulmonary disease. *Thorax* **54**, 581-586 (1999).

11 Hochstetter, J. K., Lewis, J. & Soares-Smith, L. An investigation into the immediate impact of breathlessness management on the breathless patient: randomised controlled trial. *Physiotherapy* **91**, 178-185 (2005).
12 Gift, A. G. & Narsavage, G. Validity of the numeric rating scale as a measure of dyspnea. *American Journal of Critical Care* **7**, 200-204 (1998).
13 Kolbe, J., Vamos, M., James, F., Elkind, G. & Garrett, J. Assessment of practical knowledge of self-management of acute asthma. *Chest* **109**, 86-90 (1996).
14 Jones, P. W., Quirk, F. H., Baveystock, C. M. & Littlejohns, P. A self-complete measure of health status for chronic airflow limitation. *Am Rev Respir Dis* **145**, 1321-1327 (1992).
15 Ritter, P. L., González, V. M., Laurent, D. D. & Lorig, K. R. Measurement of pain using the visual numeric scale. *The Journal of rheumatology* **33**, 574-580 (2006).
